# Effect of high compared with low dairy intake on blood pressure in overweight middle-aged adults: results of a randomized crossover intervention study

**DOI:** 10.1093/ajcn/nqz116

**Published:** 2019-06-25

**Authors:** Susan Rietsema, Coby Eelderink, Monica L Joustra, Iris M Y van Vliet, Marco van Londen, Eva Corpeleijn, Cecile M Singh-Povel, Jan M W Geurts, Jenny E Kootstra-Ros, Ralf Westerhuis, Gerjan Navis, Stephan J L Bakker

**Affiliations:** 1 Department of Internal Medicine, University Medical Center Groningen, University of Groningen, Groningen, Netherlands; 2 Interdisciplinary Center for Psychopathology and Emotion Regulation, University Medical Center Groningen, University of Groningen, Groningen, Netherlands; 3 Department of Dietetics, University Medical Center Groningen, University of Groningen, Groningen, Netherlands; 4 Department of Epidemiology, University Medical Center Groningen, University of Groningen, Groningen, Netherlands; 5 FrieslandCampina, Amersfoort, Netherlands; 6 Department of Laboratory Medicine, University Medical Center Groningen, University of Groningen, Groningen, Netherlands

**Keywords:** dairy, blood pressure, crossover study, intervention study, randomized controlled trial, dairy diet, intervention diet

## Abstract

**Background:**

Observational studies suggest that high dairy intake is associated with a lower blood pressure (BP).

**Objective:**

We aimed to investigate the effect of a high-dairy diet (HDD) as compared with a low-dairy diet (LDD) on BP in overweight middle-aged adults.

**Methods:**

Fifty-two overweight men and women were included in a randomized crossover intervention study. Each subject consumed 2 isocaloric diets for 6 wk, an LDD (≤1 dairy portion per day) and an HDD (6 or 5 reduced-fat dairy portions for men and women, respectively), with a 4-wk washout period in between the diets during which the subjects consumed their habitual diet. BP was measured at the start and at the end of the intervention diets. The effect of the intervention study was evaluated by 2-sample *t* tests. Mixed-model analyses were used for adjustment for the potential influence of changes in dietary protein and mineral intake and risk factors for hypertension including body weight and plasma cholesterol.

**Results:**

Consumption of an HDD as compared with an LDD resulted in a reduction of both systolic BP (mean ± SD: 4.6 ± 11.2 mm Hg, *P* < 0.01) and diastolic BP (3.0 ± 6.7 mm Hg, *P* < 0.01). In further analyses, these reductions appeared dependent on the concomitant increase in calcium intake.

**Conclusions:**

This intervention study shows that an HDD results in a reduction of both systolic and diastolic BP in overweight middle-aged men and women. If the results of our study are reproduced by other studies, advice for high dairy intake may be added to treatment and prevention of high BP. This trial was registered at trialregister.nl as NTR4899.

## Introduction

Hypertension is an important risk factor for various cardiovascular diseases, including stroke, coronary artery disease, heart failure, and peripheral vascular disease (1). According to the LifeLines Cohort Study, conducted in the Netherlands, 23% of people between the age of 18 and 65 y and 69% of people above the age of 65 y had hypertension in 2013 (2). According to the NHANES, 34% of people in the United States above the age of 20 y had hypertension between 2011 and 2014 (3). As a result, hypertension places an immense burden on the health care system with estimated direct and indirect costs of $53.2 billion dollars for the US in 2013 (3).

Blood pressure (BP) is largely lifestyle related and one of the ways BP can be modified is by diet. Well-established dietary modifications that lower BP are weight loss, lowering dietary salt intake, an increase of dietary potassium intake, and moderation of alcohol consumption (4). Also, strong and consistent evidence demonstrates that the Dietary Approaches to Stop Hypertension (DASH) diet, which is rich in fruits, vegetables, legumes, and low-fat dairy products, and is low in snacks, sweets, meat, and saturated and total fat, has a BP-lowering effect (4–6). A recently performed review estimated the specific contribution of dairy in the BP-lowering effect of the DASH diet to be 50% (7). However, because of differences in macronutrient composition of the various diets in the original DASH trial, this estimated contribution remains uncertain (7).

It is often suggested that dairy has an important BP-lowering effect, and that the concomitant increase in dietary intake of protein, calcium, potassium, and magnesium may mediate this effect (8–11). Accordingly, epidemiological studies are consistent in showing an inverse association between dairy intake and BP, as well as an inverse association between dairy and stroke (7). However, in 2010, the US Dietary Guidelines Advisory Committee (DGAC) concluded that there is too little evidence that supports an independent relation between the intake of dairy and BP to allow for recommendation of high dairy intake as part of a BP-lowering diet (12). Therefore, the DGAC made a request for results of randomized controlled trials (RCTs) that attempt to answer the question whether intake of dairy products alters BP.

It should be noted that for public health advice it is important that the study design is close to a real-life setting. Therefore, it is important that subjects are able to freely choose how to compose the low-dairy diet (LDD) and high-dairy diet (HDD). The aim of this study was to investigate, in an RCT close to a Dutch real-life setting, whether high dairy intake compared with low dairy intake results in a change in BP and, if so, to investigate whether and to what extent this effect can be explained by changes in intake of protein and minerals.

## Methods

### Subjects

Subjects included in the current study were healthy, overweight men and postmenopausal women aged between 45 and 65 y, with a BMI (in kg/m^2^) ≥25 to ≤30, consuming 3 main meals a day including breakfast. The subjects had a relatively stable body weight (self-reported fluctuations in body weight <3 kg in the past 3 mo) and had no intention to lose weight until the end of the study. Individuals who were involved in intensive sports activities (e.g., playing football, tennis, running, race-cycling, swimming) more than twice a week were excluded, as were those on any slimming or medically prescribed diet, those who had a vegan or macrobiotic lifestyle, or those who had a reported lactose allergy or sensitivity. Further exclusion criteria were diabetes mellitus [using the criteria of the American Diabetes Association (13): fasting glucose ≥7.0 mmol/L, glycated hemoglobin ≥6.5% (48 mmol/mol)], positive HIV, hepatitis B surface antigen and/or hepatitis C infection at screening, and clinically relevant abnormalities in blood lipids (total cholesterol >8 mmol/L, triglycerides >6 mmol/L, LDL cholesterol >5.7 mmol/L), hematology [Hb <8.7 mmol/L (male) or <7.5 mmol/L (female)], or markers for liver damage (alanine aminotransferase and aspartate aminotransferase >45 U/L) and kidney damage (urinary albumin:creatinine ratio >30 mg/mmol). Also, individuals with gastrointestinal disorders or digestive tract surgery in the past and individuals using lipid-lowering drugs (from screening until the end of the study) or antibiotics (from 1 mo before screening) were excluded. The stable use of BP-lowering medication was allowed. Intake of nutritional supplements was not allowed from screening until the end of the study.

**FIGURE 1:**
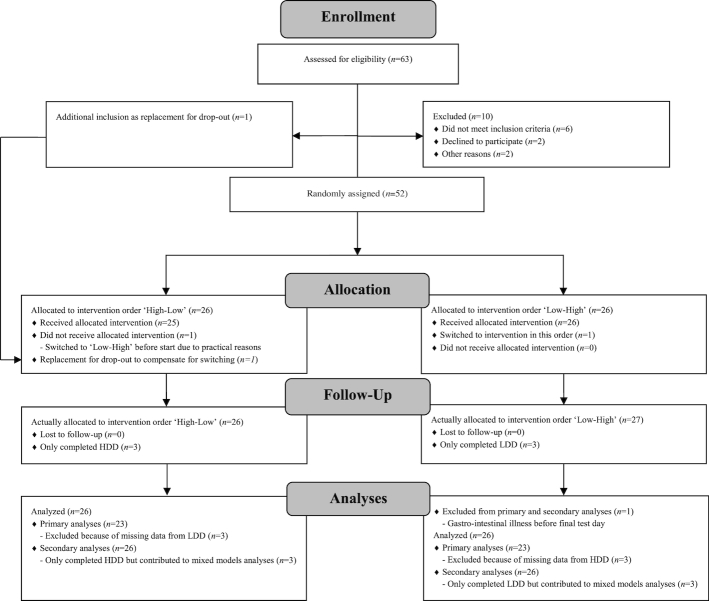
Consolidated Standards of Reporting Trials 2010 flow diagram. HDD, high-dairy diet; LDD, low-dairy diet.

Volunteers were recruited by an advertisement in a local newspaper in 2015–2016. Those interested received an information letter, Informed Consent Form (ICF), and a prescreening questionnaire. Suitable participants who returned a signed ICF and a completed questionnaire were invited for a screening visit to check for additional inclusion and exclusion criteria. Eventually 52 subjects were included and randomly assigned to a “Low-High” sequence or a “High-Low” sequence (**Figure 1**). Random assignment was based on minimization (using the software Minim, Stephen Evans, Patrick Royston and Simon Day, UK) (14) to ensure minimal differences in the “High-Low” and “Low-High” sequences based on gender (male or female), age (45–55 or 55–65 y), and BMI (25–27 or 28–30).

The study design made it impossible to blind the subjects or the researcher and dietitians to the diet. The sample analyses and data processing were blinded, where plasma samples and raw data were labeled by sequence and not by diet.

The intervention study (NTR4899) was conducted according to the principles of the Declaration of Helsinki (last amended by the 64th World Medical Association General Assembly, Fortaleza, Brazil, October, 2013) and in accordance with the Medical Research Involving Human Subjects Act [in Dutch, the Wet medisch-wetenschappelijk onderzoek met mensen (WMO)]. The study was approved by the Medical Ethics Committee of the University Medical Center Groningen (UMCG), Groningen, Netherlands (METc 2014/298).

### Study design and dietary intervention

This study is a secondary outcome analysis on BP of an RCT with crossover design conducted in the UMCG in the Netherlands. The primary outcome of the study was metabolic flexibility. Glycemic and insulinemic responses during an oral-glucose-tolerance test (OGTT) and subsequent fasting, differences in glucose kinetics during an OGTT, differences in insulin sensitivity during an OGTT, and changes in BP were secondary outcome variables in the research. Data on metabolic flexibility, on glycemic and insulinemic responses during an OGTT and subsequent fasting, on differences in glucose kinetics during an OGTT, and on differences in insulin sensitivity during an OGTT have been published (15). In a later stage, samples of the underlying biobank that was established during the study will be used for assessment of other secondary outcomes.

**FIGURE 2:**
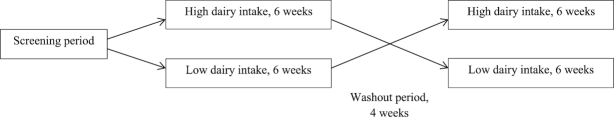
Study design.

The study consisted of two 6-wk intervention periods, an LDD (≤1 portion of dairy each day) and an HDD (6 or 5 portions of dairy each day for men and women, respectively), in random order. The 2 intervention periods were separated with a 4-wk washout period (**Figure 2**).

A portion of dairy consisted of 250 mL semiskimmed milk or butter milk, 200 g semiskimmed yogurt, or 30 g reduced-fat cheese (1 slice; maturity according to personal preference). Subjects on the HDD were instructed to consume ≥1 slice (but no more than 2 slices) of cheese and 2 portions of yogurt each day. The remaining portions of dairy were chosen by the subjects and had to consist of the described dairy products. No other dairy products were allowed during either of the intervention diets, except for the occasional use of small amounts of dairy (e.g., coffee creamer, cooking cream). All subjects were instructed to keep a food diary for 3 consecutive days, including 1 weekend day. The food diaries had to be completed before the start, and in week 3 and week 6, of each intervention diet. In addition, the subjects reported daily the number and types of dairy portions consumed. In the washout period the subjects went back to their habitual diet.

The subjects were instructed to keep a stable body weight and therefore the 2 intervention diets were intended to be isocaloric. To achieve 2 isocaloric diets, the subjects were instructed by a dietitian from the UMCG. At the start of each intervention period, the subjects attended a consultation with the dietitian to discuss the dietary assessment and to provide information about restrictions and obligations of the allocated intervention diet. Suggestions were given regarding the incorporation of a high amount of dairy into the normal diet. When necessary, the subjects were contacted by telephone, helping them to maintain their body weight or to give additional advice regarding the diet. Habitual medication had to be used in both intervention periods and was reported in the case report form. The additional use of paracetamol and any treatment for adverse events was allowed. The prescribed FrieslandCampina dairy products were purchased by the subjects at their local supermarket and the costs were reimbursed.

A dietitian calculated energy and nutrient intake from the food diaries, based on the Dutch Food Composition Database 2016, using the software program EvryDietist 6.4.2.1 (Evry BV). The calculated dietary intakes of protein, calcium, sodium, potassium, and magnesium were used as an indication of dietary intake.

### Clinical and biochemical measurements

Clinical and biochemical tests were conducted before the start of the intervention and at the end of the 6-wk period, at the visit in week 6. One day before the visit in week 6, the subjects were asked to refrain from alcohol consumption and strenuous exercise. Also, no food and drinks were allowed after 2000 the day before the visit in week 6, except for water. Subjects recorded in a diary what they had for dinner the evening before the visit in week 6 and consumed the exact same products the day before the corresponding visit in week 6 of the second intervention diet.

#### Anthropometry and total body fat percentage

Height and body weight were determined with a wall-mounted stadiometer (Seca 222, Seca GmbH) and digital weighing scale (Seca 877, Seca GmbH), respectively. Waist circumference and hip circumference were determined with a measuring tape with a standardized retraction mechanism (Seca 201, Seca GmbH). Total body fat percentage was estimated in week 6 of both intervention periods using a multifrequency Bioelectrical Impedance Analyzer (QuadScan 4000, BodyStat).

#### BP and pulse rate

BP and pulse rate were measured on the right arm with a BP monitor [Omron 705IT (HEM-759P-E2), Omron Healthcare Corporation]. Before the measurements, the subjects were positioned semiupright on an examination table and were instructed not to talk. A minimum of 2 measurements were performed and the mean of those measurements was used in the analyses.

#### Blood samples

Blood was drawn in the morning after an overnight fast for measurements of plasma glucose, insulin, triglycerides, and HDL, LDL, and total cholesterol. Blood samples were collected into 2-mL BD Vacutainer NaF tubes (BD Diagnostics Benelux) for plasma glucose, 5-mL EDTA tubes for plasma insulin, and 4.5-mL LiHep tubes for plasma triglycerides and HDL, LDL, and total cholesterol. The blood samples were then centrifuged at 1300 × *g* for 10 min at room temperature within 30 min and after that, the samples were divided into aliquots in 500-µL tubes and frozen at −80°C within 30 min after centrifugation. All blood samples were measured using standard Roche Diagnostics reagents.

#### Urine samples

One day before the visit in week 6, the subjects were asked to collect their urine for 24 h in a container and to keep it refrigerated. The volume was calculated by dividing the weight of the urine by 1.015. Aliquots were frozen in 2-mL tubes at −80°C after centrifugation at 2000 × *g* for 10 min at room temperature. The 24-h urinary excretions of urea, calcium, sodium, potassium, and magnesium were used as a marker for dietary intake. All measurements were performed on a Roche/Hitachi Modular automatic analyzer (Roche Diagnostics).

### Statistical analyses

Normally distributed variables are displayed as means ± SDs. Distributions of the data were checked using histograms and normal probability (Q-Q) plots. For lognormal distributed variables, the logarithmic values were used for statistical analyses.

Power calculation was performed based on the primary endpoint of the original study (metabolic flexibility).

To test for a carryover effect, independent-sample *t* tests were conducted to compare the sum of the outcome variables [systolic BP (SBP), diastolic BP (DBP), and pulse rate] at the end of week 6 of both intervention periods between the group of subjects that started with the LDD and the group of subjects that started with the HDD.

Primary analyses were based on complete cases by means of paired *t* tests. Secondary analyses were based on all subjects who completed ≥1 intervention period and were performed by means of mixed models. Mixed-model analyses were conducted with SBP, DBP, and pulse rate as dependent variables. The intervention, period, and the interaction between the intervention and period were treated as the main fixed effects. Age, gender, and the value of the dependent variable at baseline were added into the mixed model as fixed effects, and so were other potential confounders.

Paired *t* tests were used to test whether the dietary intake and the 24-h urinary excretions of protein/urea, calcium, sodium, potassium, and magnesium were different at the end of the LDD compared with the HDD. Because dietary intakes of protein, calcium, sodium, potassium, and magnesium are known to affect BP (8–11), and changes in dietary intakes of these might potentially explain changes in BP in response to the dietary intervention, we performed separate mixed-model analyses to which these variables were added. Protein, calcium, potassium, and magnesium intake can be well estimated from dietary questionnaires, but this is much less true for sodium, for which urinary excretion serves as the gold standard (16). For reasons of consistency, we performed separate analyses in which we adjusted for each component derived from dietary questionnaires and for each component derived from 24-h urinary excretion.

Paired *t* tests were also used to test whether plasma cholesterol parameters and specific subject characteristics, including body weight, BMI, and waist circumference, were different at the end of the LDD compared with the HDD. Because particularly body weight, BMI, and waist circumference are known to be related to BP (4, 17), and changes in these might potentially explain changes in BP in response to the dietary intervention, we performed separate mixed-model analyses in which these variables were added. Exclusion of subjects with BP-lowering medication did not materially change the results.

*P* < 0.05 was considered to be statistically significant. All statistical analyses were conducted with IBM SPSS Statistics version 23.

## Results

### Subjects

Of the 52 subjects, 44.2% were male (*n* = 23) and all of them were overweight (BMI 28.0 ± 1.9) at baseline. Twenty subjects had hypertension (SBP ≥ 140 mm Hg or DBP ≥ 90 mm Hg) at baseline and 3 subjects used BP-lowering medication throughout the entire study. The subjects had an SBP and DBP of 133.3 ± 17.1 and 82.9 ± 9.9 mm Hg, respectively, with a pulse rate of 68.0 ± 8.3/min (**Table 1**). In total, 6 subjects dropped out of the study after completion of 1 intervention diet for reasons related to the primary outcomes of the original study (1 for claustrophobia, 1 for bronchitis, 2 were sick after the OGTT, 2 were unable to continuously draw blood) and unrelated to the diet. One subject was excluded from analysis due to gastrointestinal illness before the final test day. After the random assignment, but before the start of the study, for personal practical reasons 1 subject was switched from intervention order “High-Low” to “Low-High.” To compensate for the dropouts, 1 extra subject was recruited to the “High-Low” sequence, resulting in 52 subjects who completed ≥1 intervention diet. Of these, 49 subjects completed the LDD and 49 subjects completed the HDD, resulting in 46 subjects who completed both intervention diets—and thus there were 3 subjects who only completed the LDD and 3 subjects who only completed the HDD (Figure 1). Primary analyses were based on complete cases (*n* = 46) and secondary analyses on all subjects that completed ≥1 intervention period (*n* = 52). Subject characteristics at the end of the LDD and the HDD are presented in **Table 2**. At the end of the HDD, subjects had a slightly higher body weight (84.0 compared with 83.6 kg, *P* = 0.008), and thus a slightly higher BMI (27.8 compared with 27.7, *P* = 0.007), and lower plasma HDL-cholesterol concentrations than at the end of the LDD (−0.05 mmol/L, *P* = 0.002). There was no difference in waist circumference (*P* = 0.195), total body fat (*P* = 0.212), or plasma concentrations of total cholesterol (*P* = 0.622), LDL cholesterol (*P* = 0.777), and triglycerides (*P* = 0.068) at the end of the HDD as compared with at the end of the LDD (Table 2). The calculated total energy intake was significantly higher during the HDD than during the LDD (2304 compared with 2151 kcal/24 h, *P* = 0.021) (Table 2). The same was true for the calculated dietary intakes of protein, calcium, potassium, and magnesium. Also, the 24-h urinary excretions of urea, calcium, and potassium were significantly higher at the end of the HDD than at the end of the LDD (Table 2). There was no difference in dietary intake and 24-h urinary excretion of sodium at the end of the HDD and at the end of the LDD.

**TABLE 1:** Baseline characteristics^1^

Characteristics	Values
Gender, *n* (%)	
Male	23 (44.2)
Female	29 (55.8)
Age, y	58.6 ± 4.8
SBP, mm Hg	133.3 ± 17.1
DBP, mm Hg	82.9 ± 9.9
Pulse rate/min	68.0 ± 8.3
Hypertension,^2^*n* (%)	20 (38.5)
Antihypertensive treatment, *n* (%)	3 (5.8)
Length, cm	174.0 ± 9.5
Weight, kg	85.1 ± 10.2
BMI, kg/m^2^	28.0 ± 1.9
WC, cm	95.2 ± 8.6
HC, cm	107.6 ± 4.5
Glucose, mmol/L	5.6 ± 0.5
Insulin, mU/L	11.2 ± 4.2
Total cholesterol, mmol/L	5.7 ± 0.9
HDL cholesterol, mmol/L	1.5 ± 0.4
LDL cholesterol, mmol/L	3.9 ± 0.9
Triglycerides, mmol/L	1.4 ± 0.7

1 Values are means ± SDs unless indicated otherwise, *n* = 52. Blood lipids were measured in plasma. DBP, diastolic blood pressure; HC, hip circumference; SBP, systolic blood pressure; WC, waist circumference.

2 Hypertension is defined as SBP ≥ 140 mm Hg or DBP ≥ 90 mm Hg.

**TABLE 2:** Effect of HDD versus LDD on blood pressure, body composition, blood lipids, dietary intakes, and 24-h urinary excretions^1^

	Intervention		Effect HDD as compared to LDD^2^		
Variable	LDD (*n* = 46)	HDD (*n* = 46)	∆ (*n* = 46)	95% CI	*P*
Blood pressure					
SBP, mm Hg	132.1 ± 15.7	127.5 ± 15.3	−4.6 ± 11.2	−8.0, −1.2	0.009
DBP, mm Hg	81.8 ± 9.2	78.8 ± 10.4	−3.0 ± 6.7	−5.0, −1.0	0.005
Pulse rate, per min	60.5 ± 7.4	59.9 ± 8.2	−0.7 ± 5.0	−2.2, 0.8	0.359
Body composition					
Weight, kg	83.6 ± 9.8	84.0 ± 9.8	0.4 ± 1.0	0.1, 0.7	0.008
BMI, kg/m^2^	27.7 ± 1.9	27.8 ± 1.9	0.1 ± 0.3	0.04, 0.2	0.007
WC, cm	94.8 ± 8.7	95.2 ± 8.7	0.5 ± 2.5	−0.3, 1.2	0.195
HC, cm	106.2 ± 4.9	106.2 ± 5.3	0.09 ± 2.3	−0.6, 0.8	0.800
Total body fat, %	34.3 ± 8.1	34.7 ± 8.1	0.4 ± 2.1	−0.2, 1.0	0.212
Blood lipids^3^					
Total cholesterol, mmol/L	5.3 ± 0.9	5.2 ± 0.8	−0.03 ± 0.4	−0.2, 0.09	0.622
HDL-cholesterol, mmol/L	1.4 ± 0.4	1.4 ± 0.3	−0.05 ± 0.1	−0.09, −0.02	0.002
LDL-cholesterol, mmol/L	3.6 ± 0.8	3.5 ± 0.8	−0.02 ± 0.4	−0.1, 0.09	0.777
Triglycerides, mmol/L	1.2 ± 0.5	1.2 ± 0.6	0.1 ± 0.4	−0.008, 0.2	0.068
Dietary intakes^4^					
Energy, kcal/d	2151.1 ± 461.8	2304.4 ± 589.7	153.3 ± 424.7	24.2, 282.4	0.021
Protein, g/d	78.4 ± 18.0	109.3 ± 25.6	30.9 ± 19.3	25.1, 36.8	<0.001
Calcium, mg/d	719.0 ± 157.4	1956.8 ± 283.0	1237.8 ± 303.0	1145.7, 1329.9	<0.001
Sodium, mg/d	2621.4 ± 691.6	2742.5 ± 803.3	121.2 ± 873.5	−144.4, 386.7	0.363
Potassium, mg/d	3379.9 ± 673.9	4100.2 ± 847.4	720.4 ± 809.6	474.2, 966.5	<0.001
Magnesium, mg/d	363.0 ± 99.2	409.4 ± 93.0	46.5 ± 114.0	11.8, 81.1	0.010
24-h urinary excretions, mmol/24 h					
Urea	377.1 ± 126.8	482.0 ± 121.9	104.9 ± 123.8	68.1, 141.6	<0.001
Calcium	4.1 ± 1.8	5.0 ± 2.4	0.8 ± 1.7	0.3, 1.4	0.002
Sodium	148.0 ± 63.1	145.1 ± 67.4	−2.9 ± 56.9	−19.8, 14.0	0.729
Potassium	85.8 ± 25.7	98.7 ± 27.6	12.9 ± 29.3	4.2, 21.6	0.005
Magnesium	4.7 ± 2.0	4.5 ± 1.4	−0.2 ± 2.2	−0.8, 0.5	0.563

1 Values are presented as mean ± SD. Analyses were performed based on complete cases. *P*values were obtained from paired *t*tests. DBP, diastolic blood pressure; HC, hip circumference; HDD, high dairy diet; LDD, low dairy diet; SBP, systolic blood pressure; WC, waist circumference.

2 ∆ was calculated as LDD minus HDD.

3 Blood lipids were measured in plasma.

4 Dietary intakes were calculated from food diaries.

### Intervention effect on SBP, DBP, and pulse rate

SBP and DBP were lower after the HDD than after the LDD (127.5 compared with 132.1 mm Hg, *P* < 0.01 and 78.8 compared with 81.8 mm Hg, *P* < 0.01, respectively) (Table 2). In further analyses, we investigated whether the effect of the HDD as compared with the LDD was independent of change in other variables, data on which are listed in **Supplemental Table 1**. The effect of the HDD on measured SBP and DBP remained significant when adjusted for body weight and other potential confounders (**Table 3**). Also, adjustment for the calculated intakes of energy, protein, sodium, potassium, and magnesium did not materially change the effect of the HDD as compared with the LDD on SBP and DBP (**Table 4**). The same was true for adjustment for 24-h urinary excretions of urea, sodium, potassium, and magnesium (**Table 5**). However, the effect of the HDD as compared with the LDD on SBP and DBP was nonsignificant after adjustment for the dietary intake of calcium and 24-h urinary excretion of calcium (Tables 4 and 5). The pulse rate was not different after the HDD as compared with the LDD (59.9 compared with 60.5/min, *P* = 0.36) (Table 2).

**TABLE 3:** Results of mixed-model analyses with adjustment for changes in body composition and plasma HDL cholesterol^1^

	Systolic blood pressure (*n* = 52)		Diastolic blood pressure (*n* = 52)		Pulse rate (*n* = 52)	
Model	∆ (95% CI)^2^	*P*	∆ (95% CI)^2^	*P*	∆ (95% CI)^2^	*P*
1	−7.1 (−13.0, −1.2)	0.019	−5.9 (−10.2, −1.5)	0.008	−1.7 (−5.0, 1.6)	0.312
2	−7.5 (−13.2, −1.7)	0.011	−6.0 (−10.2, −1.7)	0.007	−1.8 (−5.1, 1.4)	0.266
3	−6.7 (−12.6, −0.8)	0.028	−5.4 (−9.8, −1.1)	0.015	−1.5 (−4.8, 1.8)	0.375
4	−7.1 (−13.0, −1.3)	0.018	−5.7 (−10.0, −1.5)	0.009	−1.7 (−5.0, 1.6)	0.309
5	−6.9 (−12.8, −0.9)	0.024	−5.6 (−10.0, −1.2)	0.013	−1.5 (−4.8, 1.7)	0.353
6	−7.0 (−12.9, −1.1)	0.021	−5.8 (−10.1, −1.4)	0.010	−1.7 (−5.0, 1.7)	0.325

1 Analyses were performed with all subjects who completed ≥1 intervention diet. Model 1: adjusted for period and intervention × period, gender, age, energy intake (kcal), and baseline value of the variable. Model 2: model 1 + weight (kg). Model 3: model 1 + BMI (kg/m^2^). Model 4: model 1 + waist circumference (cm). Model 5: model 1 + total body fat (%). Model 6: model 1 + plasma HDL cholesterol (mmol/L).

2 ∆ was calculated as low-dairy diet minus high-dairy diet.

**TABLE 4:** Results of mixed-model analyses with adjustment for changes in dietary intakes^1^

	Systolic blood pressure (*n* = 52)		Diastolic blood pressure (*n* = 52)		Pulse rate (*n* = 52)	
Model	∆ (95% CI)^2^	*P*	∆ (95% CI)^2^	*P*	∆ (95% CI)^2^	*P*
1	−6.5 (−12.4, −0.7)	0.029	−5.5 (−9.8, −1.2)	0.012	−1.8 (−5.1, 1.4)	0.271
2	−7.1 (−13.0, −1.2)	0.019	−5.9 (−10.2, −1.5)	0.008	−1.7 (−5.0, 1.6)	0.312
3	−8.5 (−15.4, −1.6)	0.017	−5.7 (−10.9, −0.6)	0.030	−1.9 (−5.7, 1.9)	0.322
4	2.3 (−12.2, 16.7)	0.755	−0.8 (−11.4, 9.8)	0.883	2.1 (−6.3, 10.4)	0.623
5	−6.1 (−12.6, −0.8)	0.026	−5.5 (−9.8, −1.2)	0.013	−1.7 (−5.1, 1.6)	0.294
6	−6.9 (−13.3, −0.5)	0.034	−4.8 (−9.4, −0.1)	0.046	−0.7 (−4.3, 2.8)	0.682
7	−7.5 (−13.5, −1.5)	0.015	−5.9 (−10.3, −1.5)	0.010	−1.3 (−4.7, 2.0)	0.431

1 Analyses were performed with all subjects who completed ≥1 intervention diet. Model 1: adjusted for period and intervention × period, gender, age, and baseline value of the variable. Model 2: model 1 + energy intake (kcal/d). Model 3: model 1 + protein intake (g/d). Model 4: model 1 + calcium intake (mg/d). Model 5: model 1 + sodium intake (mg/d). Model 6: model 1 + potassium intake (mg/d). Model 7: model 1 + magnesium intake (mg/d).

2 ∆ was calculated as low-dairy diet minus high-dairy diet.

**TABLE 5:** Results of mixed-model analyses with adjustment for changes in 24-h urine excretion^1^

	Systolic blood pressure (*n* = 52)		Diastolic blood pressure (*n* = 52)		Pulse rate (*n* = 52)	
Model	∆ (95% CI)^2^	*P*	∆ (95% CI)^2^	*P*	∆ (95% CI)^2^	*P*
1	−6.5 (−12.4, −0.7)	0.029	−5.5 (−9.8, −1.2)	0.012	−1.8 (−5.1, 1.4)	0.271
2	−7.0 (−12.9, −1.1)	0.020	−5.8 (−10.1, −1.6)	0.008	−1.7 (−5.1, 1.8)	0.340
3	−4.7 (−10.7, 1.3)	0.121	−3.5 (−7.8, 0.8)	0.111	−0.7 (−3.9, 2.6)	0.691
4	−6.6 (−12.5, −0.8)	0.027	−5.5 (−9.8, −1.2)	0.014	−1.8 (−5.0, 1.5)	0.279
5	−6.6 (−12.7, −0.6)	0.032	−4.9 (−9.3, −0.5)	0.030	−0.7 (−3.9, 2.5)	0.668
6	−6.4 (−12.2, −0.6)	0.032	−5.3 (−9.5, −1.0)	0.015	−1.6 (−4.7, 1.6)	0.319

1 Analyses were performed with all subjects who completed ≥1 intervention diet. Model 1: adjusted for period and intervention × period, gender, age, and baseline value of the variable. Model 2: model 1 + 24-h urinary excretion of urea (mmol/24 h). Model 3: model 1 + 24-h urinary excretion of calcium (mmol/24 h). Model 4: model 1 + 24-h urinary excretion of sodium (mmol/24 h). Model 5: model 1 + 24-h urinary excretion of potassium (mmol/24 h). Model 6: model 1 + 24-h urinary excretion of magnesium (mmol/24 h).

2 ∆ was calculated as low-dairy diet minus high-dairy diet.

## Discussion

The aim of this study was to investigate the effect of an HDD as compared with an LDD on BP in overweight middle-aged adults. As mentioned before, BP is an important risk factor for various cardiovascular diseases (1) and, therefore, it is very important to lower BP. Previously performed prospective studies concluded that hypertension was associated with a substantial amount of avoidable deaths as well as hospitalization for coronary heart disease and stroke and, on top of that, the population attributable risk of hypertension on mortality was 2–3 times greater than that of diabetes (18). The population attributable risk of hypertension on incident heart failure was greater than those of physical inactivity, smoking, and impaired glucose control (19). These results show the importance of BP-lowering interventions.

In the primary analyses of cases with complete data, we found a significant effect of the HDD as compared with the LDD on both SBP and DBP. In secondary analyses, in which all subjects who completed ≥1 intervention diet were included, these results remained materially unchanged. In further secondary analyses, in which the use of mixed models allowed for adjustment for the potential influence of changes in calculated dietary intakes of protein, calcium, sodium, potassium, and magnesium on the effect on BP, we found no indication of mediation of the effect of the HDD on BP by higher dietary intake of either protein, sodium, potassium, or magnesium. These secondary analyses strongly support the notion that the BP-lowering effect of high dairy intake is independent of concomitant changes in other dietary components that have been implicated in BP regulation, because high dietary intakes of protein, potassium, and magnesium have been shown to have BP-lowering effects, whereas high dietary intake of sodium has been shown to have BP-raising effects (10, 20–23). The effect of the HDD on BP appeared, however, entirely dependent on the concomitant change in dietary intake of calcium. This was supported by the secondary analyses in which we adjusted for the 24-h urinary excretions of urea, calcium, sodium, potassium, and magnesium.

Interestingly, a recently performed review and meta-analysis of RCTs, investigating the effects of calcium plus vitamin D supplementation on BP, found no effect on BP (24). It can be hypothesized that calcium in combination with other minerals in dairy has a stronger effect on BP than calcium alone. This hypothesis is supported by Houston and Harper (25), who suggested that calcium in combination with other ions, including sodium, potassium, and magnesium, provides an ionic balance that results in the reduction of BP. It is also known that microbial SCFAs, which are byproducts of microbial metabolism and are formed after the consumption of yogurt, lower BP through their effect on the renin–angiotensin system (26). However, SCFAs are also produced by the fermentation of dietary fibers, which were more prominently present in the diet while subjects were on the LDD than while they were on the HDD. Alternatively, these results may be explained by the fact that dietary intake of calcium and 24-h urinary excretion of calcium are merely statistical—and not necessarily causal—intermediates between dairy intake and BP, which makes it hard to draw firm conclusions on actual causality.

This study is, to our knowledge, the first to present a reduction of both SBP and DBP after an HDD, where many other studies only found a reduction of SBP (27–29) or no reduction of BP at all (30–34). Dairy contains several components including the amino acid cysteine, that through several mechanisms, including increased synthesis of the vasodilator gas hydrogen sulfide, results in vasodilatation and may thereby have an effect on BP (35, 36). Because the production, release, and availability of nitrogen monoxide are important for vasodilatation, and because dairy is a source of cysteine and indirectly a source of nitrogen monoxide, it is possible that the established BP-lowering effect of dairy is achieved by increased dietary intake of cysteine.

Although we expected the 6-wk intervention periods to be long enough to induce an effect of dairy on BP (28), Wennersberg et al. (32) found no effect of dairy on BP, attributing that to a “short intervention period” of 6 mo. A possible explanation might be that dairy has a BP-lowering effect in the short term, whereas the body eventually regulates the BP back to its former range over a longer term. Contradicting, however, are the results of the meta-analysis of observational studies performed by Lee et al. (37), that concluded that an increased intake of 200 g dairy/d was associated with lower BP.

Strengths of the study include the crossover design, the few dropouts, and the consultations by a dietitian to increase the compliance of the subjects to the diets throughout the entire study. The real-life setting of the study design means that the results may be extrapolated to Dutch residents. Besides that, the 6-wk intervention periods were expected to be sufficiently long to induce an effect of the intervention diets on BP (28), but short enough to ensure compliance from the subjects. However, there are some limitations of the current study. Despite a 4-wk washout period, a significant interaction indicated a possible carryover effect for BP. Besides that, subjects gained weight during the HDD which is most likely due to higher energy intake, despite efforts to make the diets isocaloric. At the end of each intervention period, we performed a minimum of 2 BP measurements and used the mean of those measurements for analyses. It would have been preferable to have taken 3 BP measurements each day for several sequential days at the end of each intervention period. These limitations may have led to underestimation of the effect of dairy on BP.

In conclusion, the current literature provides evidence that an HDD reduces SBP. However, our crossover study found a reduction of both SBP and DBP after a high dairy intake, which may be explained by the fact that many studies were performed with other types of dairy products and many already performed intervention studies seem to be biased in several aspects. For example, it was unclear how data were obtained, dropout reasons were not reported, and many crossover studies did not test for a possible carryover effect and, besides that, many intervention diets were designed to achieve weight loss, which may have explained the BP-lowering effect of dairy in some studies. We also found a statistically significant reduction of plasma HDL cholesterol after the HDD, whereas changes in other blood lipids were nonsignificant.

The BP-lowering effect of dairy in our study was explained by the concomitant increase in dietary intake of calcium. The BP-lowering effect was independent of the higher dietary intakes of protein, potassium, and magnesium, and also independent of lower dietary intake of sodium.

The present study introduces dairy as a possible nourishment that may contribute to the reduction of BP. Dairy may therefore contribute to the prevention of cardiovascular diseases. However, this study is the first to suggest that dairy has a DBP-lowering effect besides the already suggested SBP-lowering effect. There is still need for further research to investigate the DBP-lowering effect of dairy. Also, potential effects on plasma HDL cholesterol require further attention. Only then can dairy be safely embedded in the prevention and treatment of both systolic and diastolic hypertension.

## Supplementary Material

nqz116_Supplemental_File

## Notes

Supported by the Public–Private Partnership Topconsortium voor Kennis en Innovatie (TKI) Agri & Food (TKI-AF-12104).

Data described in the article will be made available upon request.

Supplemental Table 1 is available from the “Supplementary data” link in the online posting of the article and from the same link in the online table of contents at https://academic.oup.com/ajcn/.

Abbreviations used: BP, blood pressure; DASH, Dietary Approaches to Stop Hypertension; DBP, diastolic blood pressure; DGAC, Dietary Guidelines Advisory Committee; HDD, high-dairy diet; ICF, Informed Consent Form; LDD, low-dairy diet; OGTT, oral-glucose-tolerance test; RCT, randomized controlled trial; SBP, systolic blood pressure; UMCG, University Medical Center Groningen.
