# Supplementary material for: Effect of high compared with low dairy intake on blood pressure in overweight middle-aged adults: results of a randomized crossover intervention study
Source: Am J Clin Nutr. 2019 Jun 25;110(2):340–8. doi: 10.1093/ajcn/nqz116 (PMC6669052; doi:10.1093/ajcn/nqz116)
Supplement: nqz116_Supplemental_File [file nqz116_supplemental_file.docx]

**ONLINE SUPPLEMENTAL MATERIAL**

**SUPPLEMENTAL TABLE 1**. Subject characteristics, dietary intake and 24 h urinary excretion at the end of the low dairy diet and high dairy diet^1^

|  | Low dairy diet  (*n* = 49) | High dairy diet  (*n* = 49) |
| --- | --- | --- |
| Gender |  |  |
| Male, *n* (%) | 20 (40.8) | 23 (46.9) |
| Female, *n* (%) | 29 (59.2) | 26 (53.1) |
| Age, y | 58.6 ± 4.4 | 58.9 ± 4.7 |
| SBP, mmHg | 132.5 ± 15.3 | 127.4 ± 15.6 |
| DBP, mmHg | 82.1 ± 9.0 | 78.7 ± 10.2 |
| Pulse rate, per min | 60.4 ± 7.4 | 60.1 ± 8.8 |
| Body composition |  |  |
| Length, cm | 173.4 ± 9.2 | 174.2 ± 9.6 |
| Weight, kg | 83.5 ± 9.5 | 84.9 ± 10.4 |
| BMI, kg/m^2^ | 27.7 ± 2.0 | 27.9 ± 1.9 |
| WC, cm | 94.2 ± 8.7 | 95.6 ± 8.7 |
| HC, cm | 106.4 ± 4.9 | 106.5 ± 5.3 |
| Total body fat, % | 34.7 ± 7.9 | 34.3 ± 8.0 |
| Glucose, mmol/L | 5.5 ± 0.4 | 5.6 ± 0.5 |
| Insulin, mU/L | 8.9 ± 3.3 | 10.1 ± 3.8 |
| Blood lipids^2^ |  |  |
| Total cholesterol, mmol/L | 5.3 ± 0.9 | 5.3 ± 0.8 |
| HDL-cholesterol, mmol/L | 1.5 ± 0.4 | 1.4 ± 0.3 |
| LDL-cholesterol, mmol/L | 3.6 ± 0.8 | 3.6 ± 0.7 |
| Triglycerides, mmol/L | 1.1 ± 0.5 | 1.3 ± 0.6 |
| Dietary intake^3^ |  |  |
| Energy, kCal/d | 2147.7 ± 457.1 | 2286.6 ± 582.3 |
| Protein, g/d | 78.6 ± 17.8 | 108.8 ± 25.1 |
| Calcium, mg/d | 724.8 ± 160.3 | 1965.2 ± 275.6 |
| Sodium, mg/d | 2647.3 ± 705.5 | 2723.5 ± 803.6 |
| Potassium, mg/d | 3377.3 ± 666.4 | 4123.2 ± 874.8 |
| Magnesium, mg/d | 362.9 ± 98.0 | 412.0 ± 92.9 |
| 24 h urinary excretion |  |  |
| Urea, mmol/24 h | 379.1 ± 125.1 | 485.2 ± 123.8 |
| Calcium, mmol/24 h | 4.1 ± 1.8 | 5.1 ± 2.5 |
| Sodium, mmol/24 h | 147.1 ± 62.5 | 149.0 ± 68.3 |
| Potassium, mmol/24 h | 84.6 ± 25.6 | 100.2 ± 28.5 |
| Magnesium, mmol/24 h | 4.6 ± 1.9 | 4.7 ± 1.7 |

^1^Data of all subjects who completed at least one intervention diet are presented, mean ± SD unless defined otherwise

^2^Blood lipids were measured in plasma

^3^Intake parameters were calculated from food diaries

DBP, diastolic blood pressure; HC, hip circumference; SBP, systolic blood pressure; WC, waist circumference
